# *CHD2* variants are a risk factor for photosensitivity in epilepsy

**DOI:** 10.1093/brain/awv052

**Published:** 2015-03-16

**Authors:** Elizabeth C. Galizia, Candace T. Myers, Costin Leu, Carolien G. F. de Kovel, Tatiana Afrikanova, Maria Lorena Cordero-Maldonado, Teresa G. Martins, Maxime Jacmin, Suzanne Drury, V. Krishna Chinthapalli, Hiltrud Muhle, Manuela Pendziwiat, Thomas Sander, Ann-Kathrin Ruppert, Rikke S. Møller, Holger Thiele, Roland Krause, Julian Schubert, Anna-Elina Lehesjoki, Peter Nürnberg, Holger Lerche, for the EuroEPINOMICS CoGIE Consortium, Aarno Palotie, Antonietta Coppola, Salvatore Striano, Luigi Del Gaudio, Christopher Boustred, Amy L. Schneider, Nicholas Lench, Bosanka Jocic-Jakubi, Athanasios Covanis, Giuseppe Capovilla, Pierangelo Veggiotti, Marta Piccioli, Pasquale Parisi, Laura Cantonetti, Lynette G. Sadleir, Saul A. Mullen, Samuel F. Berkovic, Ulrich Stephani, Ingo Helbig, Alexander D. Crawford, Camila V. Esguerra, Dorothee G. A. Kasteleijn-Nolst Trenité, Bobby P. C. Koeleman, Heather C. Mefford, Ingrid E. Scheffer, Sanjay M. Sisodiya

**Affiliations:** 1 NIHR Biomedical Research Centre Department of Clinical and Experimental Epilepsy, UCL Institute of Neurology, National Hospital for Neurology and Neurosurgery, Queen Square, London, UK; 2 Epilepsy Society, Bucks, UK; 3 Department of Paediatrics, University of Washington, USA; 4 Department of Medical Genetics Research, University Medical Centre Utrecht, The Netherlands; 5 Luxembourg Centre for Systems Biomedicine, University of Luxembourg, Esch-sur-Alzette, Luxembourg; 6 North East Thames Regional Genetics Laboratories, Great Ormond Street Hospital for Children NHS Foundation Trust, London, UK; 7 Department of Neuropaediatrics, University Medical Centre Schleswig-Holstein and Christian-Albrechts-University of Kiel, Kiel, Germany; 8 Cologne Centre for Genomics, University of Cologne, Cologne, Germany; 9 Danish Epilepsy Centre, Dianalund, Denmark; 10 Institute for Regional Health Services, University of Southern Denmark, Odense, Denmark; 11 Deptartment of Neurology and Epileptology, Hertie Institut for Clinical Brain Research, Tübingen, Germany; 12 Folkhälsan Institute of Genetics and Neuroscience Centre, University of Helsinki, Helsinki, Finland; 13 Research Programs Unit, Molecular Neurology, University of Helsinki, Helsinki, Finland; 14 Wellcome Trust Sanger Institute, Wellcome Trust Genome Campus, Hinxton, Cambridgeshire, UK; 15 Institute for Molecular Medicine Finland, University of Helsinki, Helsinki, Finland; 16 Program in Medical and Population Genetics and Genetic Analysis Platform, The Broad Institute of MIT and Harvard, Cambridge, USA; 17 Epilepsy Centre, Neurology Department, Federico II University of Naples, Naples, Italy; 18 Department of Medicine, University of Melbourne, Austin Health, Melbourne, Australia; 19 Department of Child Neurology, Paediatric Clinic, Clinical Centre Nis, Serbia; 20 Department of Paediatric Neurology, Paediatric Clinic, Al Sabah Hospital, Kuwait; 21 Neurology Department, The Children’s Hospital Agia Sophia, Athens, Greece; 22 Epilepsy Centre ‘C. Poma Hospital’, Mantova, Italy; 23 Department of Child Neurology and Psychiatry C. Mondino National Neurological Institute, Via Mondino, 2, 27100, Pavia, Italy; 24 Brain and Behaviour Department, University of Pavia, Pavia, Italy; 25 Neurophysiopathology Unit, San Filippo Neri Hospital, Rome, Italy; 26 Child Neurology, NESMOS Department, Faculty of Medicine and Psychology, Sapienza University, Rome, Italy; 27 Neurorehabilitation Unit, Department of Neuroscience and Neurorehabilitation, IRCCS, Bambino Gesu' Children's Hospital, Rome, Italy; 28 Department of Paediatrics and Child Health, School of Medicine and Health Sciences, University of Otago, Wellington, New Zealand; 29 Florey Institute of Neurosciences and Mental Health, and Department of Paediatrics, University of Melbourne, Royal Children’s Hospital, Melbourne, Australia; 30 Chemical Neuroscience Group, Biotechnology Centre of Oslo, University of Oslo, Oslo, Norway; 31 Laboratory for Molecular Biodiscovery, University of Leuven, Leuven, Belgium

**Keywords:** photosensitive, seizure, eyelid myoclonia with absences

## Abstract

Photosensitivity is a heritable abnormal cortical response to flickering light, manifesting as particular electroencephalographic changes, with or without seizures. Photosensitivity is prominent in a very rare epileptic encephalopathy due to *de novo CHD2* mutations, but is also seen in epileptic encephalopathies due to other gene mutations. We determined whether *CHD2* variation underlies photosensitivity in common epilepsies, specific photosensitive epilepsies and individuals with photosensitivity without seizures. We studied 580 individuals with epilepsy and either photosensitive seizures or abnormal photoparoxysmal response on electroencephalography, or both, and 55 individuals with photoparoxysmal response but no seizures. We compared *CHD2* sequence data to publicly available data from 34 427 individuals, not enriched for epilepsy. We investigated the role of unique variants seen only once in the entire data set. We sought *CHD2* variants in 238 exomes from familial genetic generalized epilepsies, and in other public exome data sets. We identified 11 unique variants in the 580 individuals with photosensitive epilepsies and 128 unique variants in the 34 427 controls: unique *CHD2* variation is over-represented in cases overall (*P* = 2·17 × 10^−5^). Among epilepsy syndromes, there was over-representation of unique *CHD2* variants (3/36 cases) in the archetypal photosensitive epilepsy syndrome, eyelid myoclonia with absences (*P* = 3·50 × 10^−4^). *CHD2* variation was not over-represented in photoparoxysmal response without seizures. Zebrafish larvae with *chd2* knockdown were tested for photosensitivity. *Chd2* knockdown markedly enhanced mild innate zebrafish larval photosensitivity. *CHD2* mutation is the first identified cause of the archetypal generalized photosensitive epilepsy syndrome, eyelid myoclonia with absences. Unique *CHD2* variants are also associated with photosensitivity in common epilepsies. *CHD2* does not encode an ion channel, opening new avenues for research into human cortical excitability.

## Abstract (precis)

Photosensitivity in epilepsy is common and has high heritability, but its genetic basis remains uncertain. Galizia *et al.* reveal an overrepresentation of unique variants of *CHD2* — which encodes the transcriptional regulator ‘chromodomain helicase DNA-binding protein 2’ — in photosensitive epilepsies, and show that *chd2* knockdown in zebrafish causes photosensitivity.

## Graphical abstract

**Figure:**
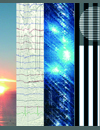
Photosensitivity in epilepsy is common and has high heritability, but its genetic basis remains uncertain. Galizia *et al.* reveal an overrepresentation of unique variants of *CHD2* — which encodes the transcriptional regulator ‘chromodomain helicase DNA-binding protein 2’ — in photosensitive epilepsies, and show that *chd2* knockdown in zebrafish causes photosensitivity.

## Introduction

Photosensitivity is a heritable abnormal cortical response to flickering light, often manifesting as EEG changes called a photoparoxysmal response (Walter *et al.*, 1946). Photoparoxysmal response may occur with seizures, and in normal subjects, or with neuropsychiatric disorders (So *et al.*, 1993). The photoparoxysmal response is age-dependent: prevalence in healthy children is between 1.4 and 8.3%, dropping to <1% in adults (Gregory *et al.*, 1993; Quirk *et al.*, 1995; Kasteleijn-Nolst Trenite *et al.*, 2003; Verrotti *et al.*, 2012). Photosensitive epilepsy is a reflex epilepsy, with seizures triggered by visual stimuli. A population-based study in Great Britain determined that the annual incidence of epilepsy with photoparoxysmal response was 1.1 per 100 000 in the overall population, and 5.7 per 100 000 between 7 and 19 years of age (Quirk *et al.*, 1995). About 40% of people with photosensitive epilepsy only have seizures on exposure to visual stimuli. Photosensitive seizures also feature in specific epilepsy syndromes, with other seizure types, and in non-syndromic epilepsies. Examples include juvenile myoclonic epilepsy (Tauer *et al.*, 2005; Koeleman *et al.*, 2013; Taylor *et al.*, 2013), other genetic generalized epilepsies (GGE) (Taylor *et al.*, 2013), idiopathic photosensitive occipital epilepsy, and other focal (Taylor *et al.*, 2004; Lu *et al.*, 2008), symptomatic occipital, and progressive myoclonic, epilepsies. The archetypal photosensitive syndrome is eyelid myoclonia with absences (EMA), a GGE characterized by rapid eyelid jerks and upward eyeball deviation on eye closure: photosensitivity is an essential feature (Sadleir *et al.*, 2012).

The photoparoxysmal response is highly heritable (Waltz and Stephani, 2000; Tauer *et al.*, 2005; Taylor *et al.*, 2013). The genetics are complex: no single gene has been implicated despite linkage to several loci and formal meta-analysis (Tauer *et al.*, 2005; De Kovel *et al.*, 2010; Verrotti *et al.*, 2012). Photosensitive epilepsies also have complex genetic architecture (Sadleir *et al.*, 2012; Taylor *et al.*, 2013), with several linked loci (De Kovel *et al.*, 2010). Photosensitivity is a trait found in many syndromes, inheritable separately from epilepsy (Newmark and Penry, 1979). It is unclear whether isolated photoparoxysmal response is a risk factor for epilepsy (De Kovel *et al.*, 2010; Verrotti *et al.*, 2012).

Photosensitivity occurs in some epileptic encephalopathies, such as Dravet syndrome due to mutation in *SCN1A* and encephalopathy associated with mutation in *CHD2* (Carvill *et al.*, 2013). Published data do not allow determination of whether the photosensitivity in these conditions is due to the underlying gene mutation or to the epileptic encephalopathy *per se*. *CHD2* encodes chromodomain helicase DNA-binding protein 2, involved in transcriptional regulation. Additional attention was drawn to *CHD2* as a candidate photosensitive epilepsy gene as the only shared gene within several reported overlapping copy number variants of the chromosome 15q26.1 region associated with complex phenotypes including epilepsy with photosensitivity. Eight patients with *de novo* deletions of 15q26 encompassing part or all of *CHD2* have been reported (Veredice *et al.*, 2009; Dhamija *et al.*, 2011; Capelli *et al.*, 2012; Lund *et al.*, 2013; Mullen *et al.*, 2013; Chénier *et al.*, 2014). We and others subsequently showed 6/500 epileptic encephalopathy cases had *de novo CHD2* mutations (Carvill *et al.*, 2013; Epi4K Consortium *et al.*, 2013; Suls *et al.*, 2013; Lund *et al.*, 2014), and recently showed that clinical photosensitivity was prominent in the rare *CHD2*-associated myoclonic encephalopathy (Thomas *et al.*, 2015).

These findings led us to hypothesize that *CHD2* disruption would be associated with common forms of photosensitive epilepsy or photosensitivity manifesting as a photoparoxysmal response alone.

## Materials and methods

Written informed consent was obtained from patients or parents/guardians for minors or those with intellectual disability. The study was approved by relevant institutional ethics committees.

We defined photosensitive epilepsy as the presence of a photoparoxysmal response (Kasteleijn-Nolst Trenité *et al.*, 2012) with a history of epilepsy, or seizures reproducibly induced by flickering light. The photoparoxysmal response *per se* was not an essential inclusion requirement in every patient with epilepsy because age, state (e.g. sleep deprivation) and antiepileptic medication affect its detectability. To test the effect of *CHD2* variation beyond the epileptic encephalopathies alone, we included a broad range of epilepsy types. Recruitment was from nine countries (see Supplementary material for details) (Tauer *et al.*, 2005; Lu *et al.*, 2008; Taylor *et al.*, 2013). The cohort included 36 patients with EMA: all had photoparoxysmal response. We sequenced *CHD2* in 580 people with photosensitive epilepsy and 55 people with photoparoxysmal response but no history of seizures. All patients were of European ancestry. The phenotypic distribution is given in Table 1.

**Table 1:** Distribution of cases by continental origin and broad syndromic classification

	Syndrome			
Cohort	GGE	Focal	Other	PPR without epilepsy
European	249	24	32	55
Australian	230*	35*	11	0
Total	479*	59*	43	55

European includes epilepsy cases from Germany (90), Italy (82), The Netherlands (75), Greece (34), Serbia (17), UK (5) and Denmark (2).

GGE = genetic generalized epilepsies, including GGE for which other information was not available, and, where classified, juvenile myoclonic epilepsy, juvenile absence epilepsy, childhood absence epilepsy, early-onset absence epilepsy, epilepsy with myoclonic atonic seizures, epilepsy with generalized tonic-clonic seizures only, and EMA.

Focal includes all types of focal epilepsies, including idiopathic photosensitive occipital lobe epilepsy (IPOE). *One Australian patient evolved from a GGE to a focal epilepsy.

Other includes Lennox-Gastaut syndrome, epilepsy due to tuberous sclerosis, epilepsy with electrical status epilepticus in sleep and epilepsies otherwise unclassified: none of these particular cases had unique *CHD2* variants.

We evaluated data from two additional exome-sequenced cohorts of GGE patients, to determine the role of *CHD2* variation in GGE *per se*, independent of photoparoxysmal response. Not all patients in these cohorts had been formally assessed for photoparoxysmal response. These two groups were the Complex Genetics of Idiopathic Epilepsies Consortium (CoGIE) cohort of 238 probands with familial GGE (Supplementary material), and a published cohort of 118 patients with GGE (Heinzen *et al.*, 2012).

Targeted sequencing of *CHD2* was undertaken either using Illumina TruSeq Custom Amplicon™ (TSCA) or molecular inversion probes (see Supplementary material for details). Whole exome sequencing (Supplementary material) was performed on five EMA samples. Coverage data for all experiments are provided in the Supplementary material. Only variants confirmed by a second method (Sanger sequencing or a second independent molecular inversion probe capture, see Supplementary material) were used in analyses.

The Exome Aggregation Consortium (ExAC) formed a large control population of disease and population genetic studies (ExAC, Cambridge, USA; URL: http://exac.broadinstitute.org accessed October 2014; non-Finnish European samples only used), giving the best available population frequency of *CHD2* variants of interest. Detailed phenotypic data are not available for these individuals; some might, if tested, have or have had photoparoxysmal response or a history of photosensitive seizures. These unselected cases are unlikely to harbour more than the best estimates of photoparoxysmal response prevalence in the general population (1.4%) (Kasteleijn-Nolst Trenite *et al.*, 2003).

We focused on unique variants, in our cohort and in ExAC: this is a well-established approach (Carvill *et al.*, 2013; Cnossen *et al.*, 2014; Wain *et al.*, 2014). We hypothesized an over-representation of unique variants in our cohort compared with the phenotypically-unselected ExAC cohort. We defined unique variants as those that occurred in one individual only, in cases and controls (from ExAC) considered together, that were non-synonymous, splice-site or frameshift. We used several methods for prediction of the functional consequences of unique variants in cases (Supplementary material). We defined ‘rare’ variants as those with a minor allele frequency <1% in the non-Finnish European ExAC samples.

We undertook functional studies. To test functional consequences of Chd2 loss in zebrafish, we used the *chd2* E2I2 morpholino reported previously (Suls *et al.*, 2013). Briefly, morpholino (12 ng) was microinjected into 1- to 2-cell-stage embryos of the AB (wild-type) strain. Embryos were raised in a dark incubator. At 1 day post-fertilization (dpf), embryos were prepared using the least possible amount of light. In parallel, control non-injected embryos from the same clutch of eggs were processed in the same manner. At 4 dpf, optic tectal field recordings were performed (Suls *et al.*, 2013) (Supplementary material). The first 10 s of recording were performed in minimal light in order to place the needle. Immediately following these first 10 s, recordings were performed in the dark for five minutes. At the end of this 5-min period, a very bright light was switched on (‘light ON’ state; six times the standard brightness level used for needle placement), and recording continued for 5 min. A paroxysm of high-frequency activity (200–500 Hz) with amplitude >3 times background level, either spontaneous or evoked by light, was defined as a polyspiking episode.

### Statistics

We performed a two-tailed Fisher’s exact test to determine whether the burden of unique variants in our case cohorts was greater than expected compared to ExAC controls. We examined the frequency of all rare variants in the entire cohort, and the frequency of unique variants only separately in patients with EMA, patients with GGE excluding EMA, and patients with focal epilepsies. The threshold for significance was set at *P* < 0·01, applying Bonferroni correction for these five comparisons. For the single separate comparison of cases with photoparoxysmal response without epilepsy and ExAC, significance was set at *P* < 0·05. For zebrafish data, comparison of the parameters of spiking activity (dark versus light condition) for each treatment group was performed using the Mann-Whitney test.

## Results

**Figure 1:**
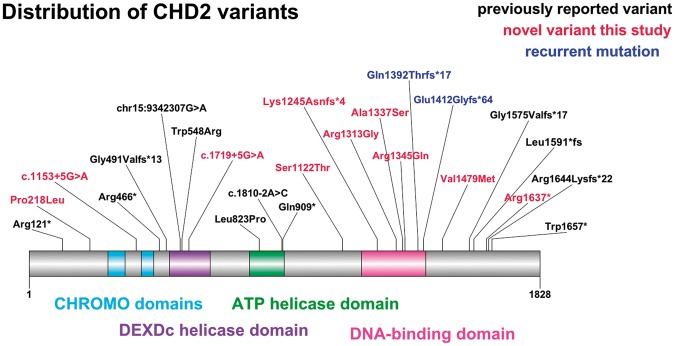
**Schematic of *CHD2* illustrating its functional (chromo, DEXDc, DNA-binding and ATP helicase) domains, the location of previously-reported variants and the unique variants in both cases and controls identified in this study**.

We identified 22 rare variants (Supplementary Table 1) in the cohort of patients with photosensitive epilepsy: 11 were unique (Table 2). There was a significant difference (*P* = 2.17 × 10^−5^) in unique variant frequency between cases (11/580 cases; 11/1160 alleles; 0.95%) and controls (128/68 854 alleles; 0.19%). The unique variants in the cases were all well covered in ExAC controls (Supplementary material). The 11 unique variants in cases were also absent from additional data sets: Exome Variant Server (http://evs.gs.washington.edu/EVS/), 1000 Genomes data set (http://www.1000genomes.org/), and dbSNP (http://www.ncbi.nlm.nih.gov/SNP). There was no difference in the overall burden of rare *CHD2* variants in cases compared to controls [22/1160 alleles (1.90%) versus 1236/68854 alleles (1.80%) respectively; *P* = 0.74]. We provide data on the frequency of variants in *CHD2* in cases and controls according to various thresholds in the Supplementary Table 2. Figure 1 shows all previously-reported variants and all unique variants identified in our cases.

**Table 2:** Patients found to have unique mutations in *CHD2* and their clinical phenotypes

Case ID	Position (NCBI.37)	Consequence	cDNA change	Protein change	Computational Analysis Score (PolyPhen-2; SIFTindel; SIFT; splice-site inference)	CADD scores (PHRED scaled)	Syndromic diagnosis	Comments
1	15:93545502	Frameshift deletion	c.4233_4236del	p.E1412Gfs*64	Deleterious (0.858)	44	GGE	
2	15:93487750	Splice site	c.1153+5G>A	NA	No change in donor site	8.124	Unclassified	
3	15:93541780	Missense	c.C3937G	p.R1313G	Probably damaging (0.98)	16.9	Unclassified	
4	15:93543742	Missense	c.G4009T	p.A1337S	Benign (0.001)	8.728	IPOE	
5	15:93496808	Splice site	c.1719+5G>A	NA	Loss of donor site	15.74	Unclassified	Learning disability
6	15:93528855	Missense	c.G3365C	p.S1122T	Benign (0.01)	4.373	GGE	
7	15:93540316	Frameshift deletion	c.3725delA	p.K1245Nfs*4	Deleterious (0.858)	43	EMA	Autism; nephrolithiasis; migraine; scoliosis
8	15:93545442	Frameshift insertion	c.4173dupA	p.Q1392Tfs*17	Deleterious (0.85)	38	EMA	*De novo* mutation
9	15:93482909	Missense	c.C653T	p.P218L	Probably damaging (0.99)	21.3	EMA	Inherited from unaffected mother
10	15:93543767	Missense	c.G4034A	p.R1345Q	Possibly damaging (0.8)	33	JME	
11	15:93563244	Nonsense	c.C4909T	p.R1637X	Probably damaging (nonsense)	49	Phenotype evolved from early-onset absence epilepsy to IPOE	*De novo* mutation
i	15:93552396	Missense	c.G4435A	p.V1479M	Probably damaging (0.996)	27.9	PPR; febrile seizures only; no epilepsy	

IPOE = idiopathic photosensitive occipital epilepsy; JME = juvenile myoclonic epilepsy; PPR = photoparoxysmal response.

We investigated the predicted deleteriousness of the 11 unique variants in the cases (Table 2). Eight of 11 unique variants (73%) had scaled CADD scores >10, placing them in the top 10% most deleterious single nucleotide variants; as a group, the 11 variants had a mean scaled CADD score of 32.6, ranking higher than 99.95% of all possible human single nucleotide variants (Kircher *et al.*, 2014).

Next, we analysed variation by epilepsy type. The archetypal photosensitive GGE syndrome EMA had the highest frequency of unique variants, found in 3/36 patients, more than expected compared to ExAC controls (3/72 alleles versus 128/68 854 alleles) (*P* = 3.50 × 10^−4^). As a *post hoc* comparison, the frequency of unique variants (4.2%) in the small EMA group is considerably greater than in our overall cohort excluding EMA (0.74%) (*P* = 0.026). Notably, two of three EMA variants were frameshift, compared to 9/128 unique variants in ExAC. One EMA variant was shown to have arisen *de novo*, strengthening its role in causation of the phenotype.

For all GGE excluding EMA, we found no significant difference compared to ExAC (4/888 alleles versus 128/68 854 alleles, *P* = 0.089). We also did not find significant over-representation in focal epilepsies compared with ExAC (2/118 alleles versus 128/68 854 alleles; *P* = 0.021). One case was included in both GGE and focal epilepsy cohorts, as the phenotype evolved from early-onset absence epilepsy to idiopathic photosensitive occipital epilepsy (Patient 11, Table 2). One of 55 (1.82%) individuals with photoparoxysmal response but no seizures had a unique *CHD2* variant (Table 2 and Fig. 1): this did not represent over-representation compared to ExAC (1/110 alleles versus 128/68 854 alleles; *P* = 0.186). This case has not developed epilepsy by the age of 18 years. We provide 99% confidence intervals (CI) (accounting for multiple comparisons) for all these comparisons in Table 3.

**Table 3:** Odds ratio for association with unique variants in *CHD2* by phenotype, with 99% CI

	*P*-value (Fisher’s exact; 2-tailed)	Odds ratio	Lower bound of 99% CI	Upper bound of 99% CI
Whole photosensitive epilepsy cohort	2.17 × 10^−5^	5.18	2.29	11.74
EMA alone	3.50 × 10^−4^	24.36	5.06	117.38
GGE excluding EMA	0.089	2.44	0.65	9.08
Focal epilepsies	0.021	9.40	1.45	61.01
Cases with PPR only	0.186	4.96	0.36	67.74

The associations with photosensitive epilepsy overall and with EMA alone are significant, as documented in the text. PPR = photoparoxysmal response.

To investigate whether *CHD2* may be associated with the broader phenotype of GGE rather than photosensitive epilepsies specifically, we tested whether rare variants in *CHD2* were enriched in patients with GGE, with or without photoparoxysmal response. Of 238 CoGIE GGE probands (Supplementary material), none had unique *CHD2* variants (not seen in ExAC or our cases). There were no unique mutations in *CHD2* in a previously-published cohort of 118 patients with GGE (Heinzen *et al.*, 2012).

**Figure 2:**
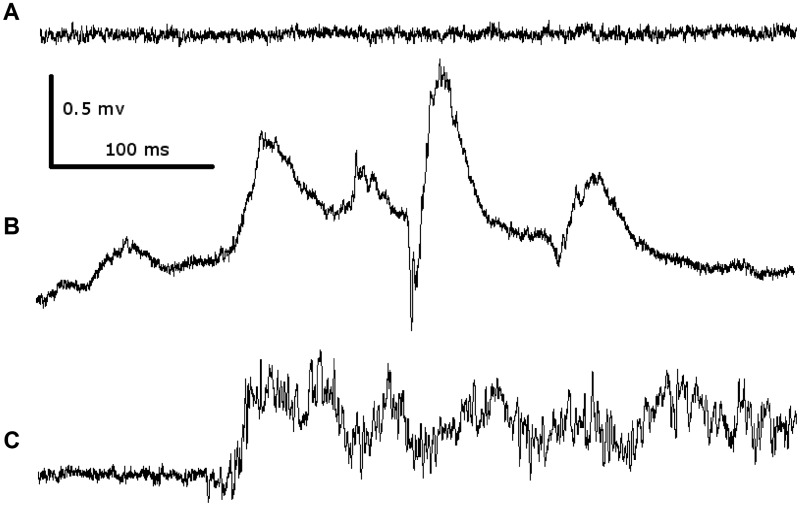
**Representative tectal field recordings of 4-dpf zebrafish larvae.** Background fragment of non-treated wild-type control in the dark (**A**); reaction of a non-injected fish to light ON - movement artefacts (wavy background) and a very short spike were observed (**B**); response to light ON of the morpholino-injected larvae: significantly more spiking activity is seen (**C**). The scale is the same for all three fragments.

To test functional consequences of Chd2 loss in zebrafish, we used the *chd2* E2I2 morpholino reported previously (Suls *et al.*, 2013). As described, *chd2 *morpholino-injected larvae displayed body curvature, excessive body pigmentation, and developmental delay (Suls *et al.*, 2013). This phenotype was observed after 50% knockdown of *chd2*. All non-treated larvae appeared normal. Recordings were obtained from 15 morpholino-injected larvae and 10 sibling controls. In comparison to 7 dpf larvae (Afrikanova *et al.*, 2013), spikes from 4 dpf larvae were shorter in duration and displayed a higher frequency of oscillations in polyspike complexes. Due to these differences, spontaneous spiking in controls was not excluded, but also quantified. We analysed duration of discharges, number of discharges under light conditions, cumulative duration of spiking activity, and cumulative discharge frequency distribution. Representative recordings are shown in Fig. 2.

**Figure 3:**
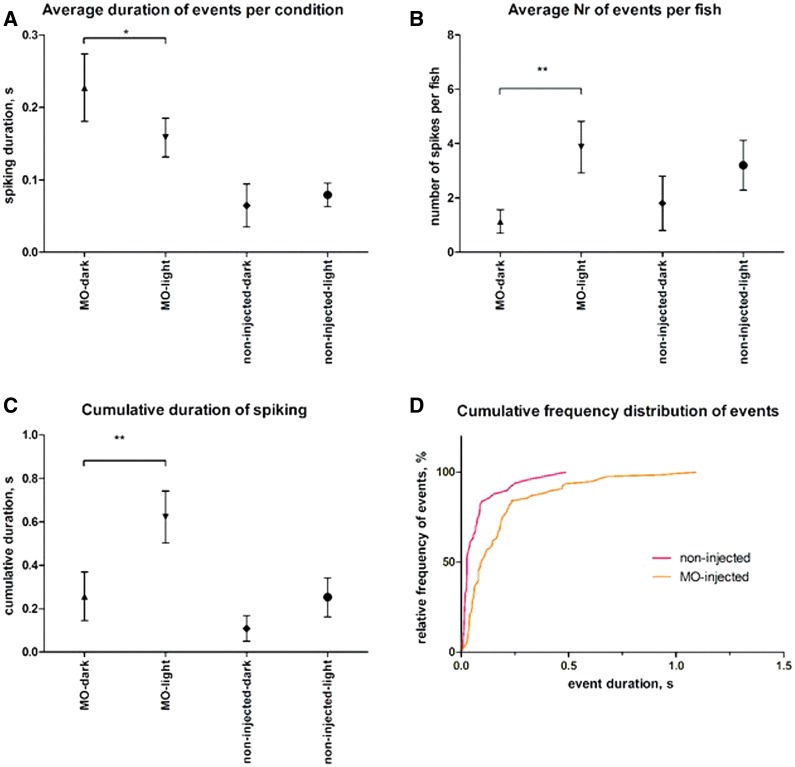
**Electrographic activity of zebrafish larvae with *chd2* knockdown and light ON stimulus.** Zebrafish larvae (4 dpf) were kept in the dark (or darkened environment, if not possible otherwise) for all groups in Danieau’s medium. Tectal field recordings were performed for the first 5 min in the dark and subsequently in light ON state for the following 5 min in morpholino-injected larvae (*n* = 15) and non-injected larvae (*n* = 10). A spiking episode, either spontaneous or evoked by light, was defined as a paroxysm of high-frequency (200–500 Hz) activity with the amplitude exceeding three times the background. Average duration of spiking events ± SEM detected per condition is shown in **A**. Average number of events per fish ± SEM is shown in **B**. Cumulative duration of spiking activity per fish as seconds ± SEM is shown in **C**. Cumulative frequency distribution of spiking episodes is shown in **D**: morpholino-injected larvae show more activity than any of the non-injected controls, and a higher photosensitivity (curve shift to the right in the light compared to the dark recordings). *P < 0.05 and **P < 0.01 Mann-Whitney test.

In line with the previous findings (Suls *et al.*, 2013), the morpholino-injected larvae showed spontaneous abnormal burst discharges. There was a preferential occurrence during the light ON state (17 discharges in the dark versus 59 in the light). In the morpholino-injected group, 14/15 larvae had discharges during the light ON state; 7/15 larvae had spiking only during the 5-min light ON state, and 10/15 showed spiking activity within the first 3–5 s after the light ON. The average duration of any event (spike or polyspike discharge) in the morpholino-injected group fell during the light ON state (Fig. 3A), attributable to the fact that morpholino-injected larvae also displayed spontaneous polyspike discharges in the dark: the events under light conditions were more heterogeneous (i.e. spontaneous polyspikes plus light-induced spiking), explaining reduced average duration. The average number of events/larva significantly increased in the morpholino-injected group in the light opposed to the dark period; this was not seen in the control group (Fig. 3B). A similar pattern was observed for cumulative duration of spiking activity (Fig. 3C): morpholino-injected larvae showed a steep increase in polyspike discharges in the light ON state, not observed for controls. The larvae from the non-injected control group also reacted to the light ON state by displaying an initial locomotor response, with 7/10 displaying short spontaneous burst activity within 2–13 s after the light was switched on. However, the overall distribution of event duration is different from that of morpholino-injected larvae (Fig. 3D): the controls’ curve lies to the left of the morpholino-injected curve, indicating that the proportion of longer discharges is higher in the morpholino-injected group.

## Discussion

We show an enrichment of unique variants in *CHD2* with photosensitivity in the common epilepsies overall, identifying *CHD2* as a photosensitive epilepsy gene. We also examined the distribution of unique variants by syndrome. *CHD2* is also the first gene to be discovered for EMA, the archetypal photosensitive epilepsy syndrome. In *CHD2* encephalopathy, though published phenotypes can be difficult to interpret, the seizure type of absence seizures with eyelid myoclonia, rather than the epilepsy syndrome, is seen in as many as 8/23 (35%) patients with *de novo CHD2* mutation or deletion (Veredice *et al.*, 2009; Dhamija *et al.*, 2011; Capelli *et al.*, 2012; Carvill *et al.*, 2013; Chénier *et al.*, 2014; Lund *et al.*, 2014). Together, these results suggest that *CHD2* is an important contributor to both the absence seizures with eyelid myoclonia seizure type and EMA epilepsy syndrome. For other epilepsy syndromes, *CHD2* variation over-representation in the photosensitive GGE or the mixed cohort of photosensitive focal epilepsies failed to meet the corrected threshold for significance. A single unique *CHD2* variant was found in one patient with photoparoxysmal response without seizures. In view of the comparatively small sizes of these syndrome cohorts, we can only confidently exclude effects with odds ratios greater than the upper limit for the 99% confidence intervals given in Table 3. Further studies in larger cohorts of these phenotypes would seem warranted.

Previous studies of photoparoxysmal response support a model of significant genetic heterogeneity and an overall complex genetic architecture (Sadleir *et al.*, 2012; Verrotti *et al.*, 2012; Taylor *et al.*, 2013): indeed, none of the several linkage regions contain *CHD2*. Our findings confirm heterogeneity and complexity in the genetics of photosensitivity, but also suggest a single gene may contribute to photosensitivity in some cases. Two mutations we detected are recurrent: p.Glu1412Glyfs*64, previously reported in epileptic encephalopathy with marked photosensitivity (Carvill *et al.*, 2013); and p.Gln1392Thrfs*17, in Lennox-Gastaut syndrome with photosensitivity (Lund *et al.*, 2014). The unique variants detected are, as a group, predicted to be amongst the most deleterious variants possible (Kircher *et al.*, 2014) and *CHD2* is amongst the genes least tolerant of functional variation (Petrovski *et al.*, 2013; Residual Variation Intolerance Score 2.37).

*CHD2* does not encode an ion channel, opening up new avenues for research into cortical excitability. *CHD2* is one of nine genes from a highly-conserved protein family with a unique domain combination: two N-terminal chromatin-organization modifier (chromo), SNF2-related helicase/ATPase and DNA-binding domains (Woodage *et al.*, 1997; Schuster *et al.*, 2002; Kulkarni *et al.*, 2008). *Chd2* knockdown zebrafish have multiple developmental abnormalities, abnormal movements and epileptiform discharges (Suls *et al.*, 2013). Disruption of *Chd2* in mice causes embryonic death in some heterozygote pups and a complex phenotype including growth retardation and lordokyphosis (Marfella *et al.*, 2006; Kulkarni *et al.*, 2008): epilepsy has not yet been described. Interestingly, the reported human mutations do not cluster to accessory domains of the protein and no obvious pattern has emerged. Recent data demonstrated that the N-terminal region of *CHD2* plays an inhibitory role, reducing DNA affinity and ATPase activity which may confer specificity, while the C-terminus enhances DNA binding and stimulates ATPase activity (Liu *et al.*, 2015). Additional studies investigating protein interacting partners and post-translational modifications of CHD2 will be necessary to understand how abnormal *CHD2* leads to photosensitive epilepsy.

Our zebrafish data show that partial (50%) loss of *chd2* function causes photosensitivity. Although Suls *et al.* (2013) showed *chd2* knockdown could cause seizures, photosensitivity was not studied. Although normal zebrafish show complex sensitivity to light (Moore and Whitmore, 2014), and untreated larvae show minor sensitivity to sudden exposure to light, morpholino-injected larvae show significantly more spiking activity on sudden light exposure. Photosensitivity on constant, rather than only flickering, light exposure has been described in humans (Oguni *et al.*, 2001). The functional consequences of each of the human mutations we detected is not known, but some at least very probably lead to loss of function, as caused by partial *chd2* knockdown that results in markedly enhanced photosensitivity in zebrafish. Together, these data strongly suggest that some human *CHD2* mutations cause photosensitivity.

There are potential limitations of our work. Different sequencing platforms were used for the various studied groups. However, we note that all unique variants in cases were confirmed by a second method, whereas for ExAC controls we used a liberal threshold to maximize sensitivity to unique variants, such that a proportion of variants selected from ExAC will be false positive: the net result of this overall conservative approach is only to reduce study power. The ExAC cohort is also the biggest relevant control data set available, and the most likely of any existing data set to provide an accurate estimate of the true frequency of unique variation in *CHD2* in a population not enriched for photosensitive epilepsy. Taking all these factors into account, the use of different platforms is very unlikely to have generated false positive results—indeed, we are more likely to have underestimated unique variant numbers in cases. It is also possible that our choice of statistical test may have missed a true association between rare variation in *CHD2* and GGE (irrespective of photoparoxysmal response or photosensitivity), and we did not test whether *CHD2* variation contributes to epilepsy more broadly: we therefore cannot exclude the possibility that rare *CHD2* variation contributes to epilepsy *per se*. Lack of parental samples meant we could only confirm variants were *de novo* in two patients. Family samples were only available in one other case (Case 9): the variant was inherited from a clinically-unaffected mother in whom no EEG studies had been carried out.

Our results provide evidence for a specific gene in a particular trait in epilepsy. Understanding the genetic basis of the photosensitivity trait is a first step to elucidating the biology that underlies photoparoxysmal response and its relation to epilepsy. Human photosensitive epilepsy paradigms have facilitated epilepsy treatment discoveries (French *et al.*, 2014): understanding photoparoxysmal response biology may increase the value of these paradigms. Our findings may also provide new directions for understanding human cortical excitability.

## Abbreviations

dpf: day post-fertilization
EMA: eyelid myoclonia with absences
ExAC: Exome Aggregation Consortium
GGE: genetic generalized epilepsy
